# Supplementary material for: Effect of Chia (Salvia hispanica L.) Associated with High-Fat Diet on the Intestinal Health of Wistar Rats
Source: Nutrients. 2022 Nov 21;14(22):4924. doi: 10.3390/nu14224924 (PMC9696280; doi:10.3390/nu14224924)
Supplement: Supplementary file 1 [file nutrients-14-04924-s001.zip › nutrients-2036154-supplementary.pdf]

Table S1. Sequencing data at the end of 35 days of treatment, according to each experimental group.

Groups	Good's coverage	Raw sequences	After filtering and cleaning		After normalization	
		Reads	Reads	OTUs	Reads	OTUs
SD	0.998 ± 0.000	29399 ± 3650	22232 ± 2825	316 ± 24	15324 ± 7	316 ± 24
SD+chia	0.997 ± 0.001	35042 ± 5041	27337 ± 3937	404 ± 51	15335 ± 39	394 ± 67
HFD	0.997 ± 0.000	25994 ± 5565	20763 ± 3619	300 ± 78	15318 ± 8	300 ± 78
HFD+chia	0.997 ± 0.000	31449 ± 7733	24569 ± 5404	396 ± 78	15313 ± 32	383 ± 69

Values referring to means ± SD, n = 8/group. SD: standard diet; SD+chia: standard diet + chia; HFD: high fat diet; HFD+chia: high fat diet + chia.

Table S2. Significantly different families and genus identified prior to FDR correction, at the end of 35 days of treatments.

	SD	SD+chia	HFD	HFD+chia
Lachnospiraceae	27.34 ± 3.49^{a}	26.61 ± 1.50^{a}	22.78 ± 1.89^{b}	26.97 ± 1.92^{a}
Muribaculaceae	9.68 ± 1.39^{b}	12.88 ± 2.96^{a}	12.63 ± 1.73^{a}	11.98 ± 1.76^{ab}
Roseburia	0.42 ± 0.14^{b}	0.68 ± 0.21^{a}	0.48 ± 0.14^{ab}	0.71 ± 0.24^{a}

Values refer to mean relative abundance ± SD, n = 6/group. SD: standard diet; SD+chia: standard diet + chia; HFD: high fat diet; HFD+chia: high fat diet + chia. ^{a,b} Treatment groups not indicated by the same letter are significantly different ($p<0.05$).
